# High precision *Neisseria gonorrhoeae* variant and antimicrobial resistance calling from metagenomic Nanopore sequencing

**DOI:** 10.1101/gr.262865.120

**Published:** 2020-09

**Authors:** Nicholas D. Sanderson, Jeremy Swann, Leanne Barker, James Kavanagh, Sarah Hoosdally, Derrick Crook, The GonFast Investigators Group, Teresa L. Street, David W. Eyre

**Affiliations:** 1 Nuffield Department of Clinical Medicine, University of Oxford, John Radcliffe Hospital, Oxford OX3 9DU, United Kingdom;; 2 National Institute for Health Research Oxford Biomedical Research Centre, John Radcliffe Hospital, Oxford OX3 9DU, United Kingdom;; 3 Big Data Institute, University of Oxford, Oxford OX3 7LF, United Kingdom

## Abstract

The rise of antimicrobial-resistant *Neisseria gonorrhoeae* is a significant public health concern. Against this background, rapid culture-independent diagnostics may allow targeted treatment and prevent onward transmission. We have previously shown metagenomic sequencing of urine samples from men with urethral gonorrhea can recover near-complete *N. gonorrhoeae* genomes. However, disentangling the *N. gonorrhoeae* genome from metagenomic samples and robustly identifying antimicrobial resistance determinants from error-prone Nanopore sequencing is a substantial bioinformatics challenge. Here, we show an *N. gonorrhoeae* diagnostic workflow for analysis of metagenomic sequencing data obtained from clinical samples using R9.4.1 Nanopore sequencing. We compared results from simulated and clinical infections with data from known reference strains and Illumina sequencing of isolates cultured from the same patients. We evaluated three Nanopore variant callers and developed a random forest classifier to filter called SNPs. Clair was the most suitable variant caller after SNP filtering. A minimum depth of 20× reads was required to confidently identify resistant determinants over the entire genome. Our findings show that metagenomic Nanopore sequencing can provide reliable diagnostic information in *N. gonorrhoeae* infection.

Antimicrobial-resistant *Neisseria gonorrhoeae* is a major public health threat, with only limited treatment options available (Unemo 2015). We have recently described that rapid long-read sequencing using the Oxford Nanopore Technologies (ONT) R9.4.1 platform offers the potential to detect and sequence near-complete *N. gonorrhoeae* genomes directly from urine samples (Street et al. 2020). This clinical metagenomic approach has the advantage that it does not require prior bacterial culture, which typically adds two to three days to diagnostic workflows and may not be available in all cases, particularly in settings where diagnostics are based on molecular testing alone. With analysis possible during sequencing (Sanderson et al. 2018), it could potentially offer a same day diagnostic tool for gonorrhea infection that can guide antimicrobial treatment.

ONT data have several potential advantages in addition to speed and the portability of the diagnostic platform. The long reads generated can allow taxonomic classification with greater specificity than is possible with short reads (Cuscó et al. 2018). Additionally, as reads containing antimicrobial resistance determinants (with the exception of those on plasmids) contain greater amounts of genetic context than is found with short reads, assignment of resistance determinants to a species is more precise. However, ONT data contain a substantial per base error rate of up to 10% with assemblies containing open reading frame disrupting insertion or deletion errors (Watson and Warr 2019). Generation of hybrid assemblies with short-read data to mitigate the error rate (De Maio et al. 2019) negates the speed and portability available with ONT. If Nanopore sequencing is to be used alone for pathogen sequencing applications directly from clinical samples, for example, for antimicrobial resistance prediction and transmission tracking, then this needs to be overcome.

Previous work (Golparian et al. 2018) shows that Nanopore 2D–based sequencing of *N. gonorrhoeae* isolates can be used to identify drug resistance determinants and to undertake phylogenetic inference. However, this work was undertaken on isolates, rather than clinical samples directly, and Nanopore 2D sequencing has since been deprecated. This study (Golparian et al. 2018) also found some differences between the phylogenies obtained from ONT and Illumina sequencing of the same isolates as a result of differences in consensus sequences called by the two methods. Most of the previous work optimizing consensus sequence calling from Nanopore data has been undertaken following viral sequencing, for example, of Ebola using Nanopolish (Quick et al. 2016) or frequency and strand bias (Grubaugh et al. 2019). Some investigators have successfully transferred these approaches to bacterial sequence data, for example, *Escherichia coli* using an optimized application of the GATK package (Greig et al. 2019).

Here, we build on this work by releasing a packaged workflow for analysis of 1D R9.4.1 Nanopore data from *N. gonorrhoeae* obtained from direct sequencing of clinical samples. To generate a whole-genome consensus sequence, we use a variant calling approach from aligned reads. For resistance determinant detection, we adopt multiple approaches including analyzing reads aligned to specific genes. For more diverse genes, we use assembled contigs to first select a reference gene before undertaking alignment.

## Results

Data from ONT (Table 1) sequencing of five *Neisseria gonorrhoeae*–containing samples were used for initial method development: three metagenomic sequences of urine samples spiked with known reference strains (WHO F, V, and X) and two from sequencing of isolates (WHO Q and H18-208). The median sequencing depth was >100× for each sample, and coverage breadth was 97%–99.7% at 1× coverage or higher (Supplemental Fig. S1). Each sequence was subsampled to varying depths between 2× and 100×.

**Table 1.**
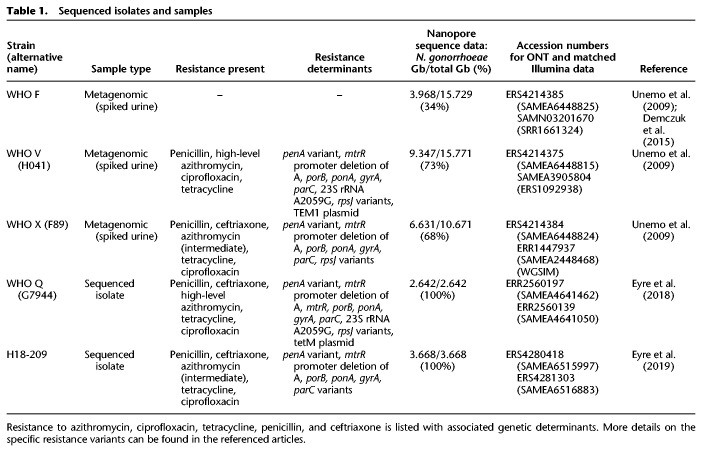
Sequenced isolates and samples

### Tuning variant calling

**Figure 1.**
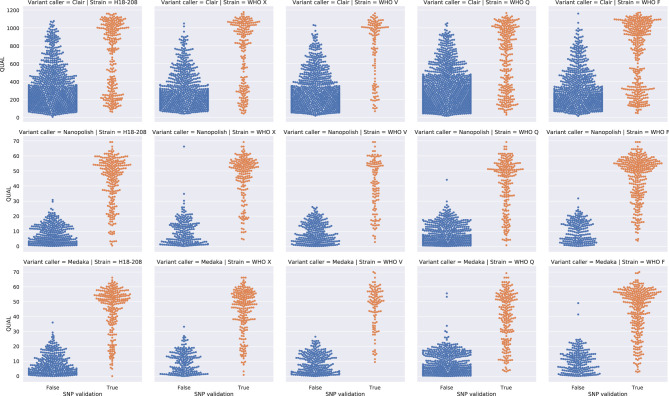
Detection of SNPs using QUAL scores alone. Swarm plots of true (orange) and false SNPs (blue) detected by Clair (*top*), Nanopolish (*middle*), and Medaka (*bottom*). Each column is a different sequence. Each row has different *y*-axis values.

Variants were called for each subsampled genome using Nanopolish, Clair, and Medaka. Previous Illumina sequences of the same isolates were used as a “truth set” or “gold standard” (Supplemental Table S1). All three variant callers identified numerous false positive SNPs compared to the Illumina data. Variant caller reported QUAL scores were unable to reliably differentiate false and true SNPs (Fig. 1), for example, using Nanopolish and a QUAL score cutoff of ≥25 for calling variants, at 100× coverage, recall was 0.94–0.97, precision 0.68–0.99, and number of false SNPs 32–1870 across the five genomes. Recall, precision, and false positive rates for Medaka and Clair were even worse (Fig. 1; Supplemental Table S2).

**Figure 2.**
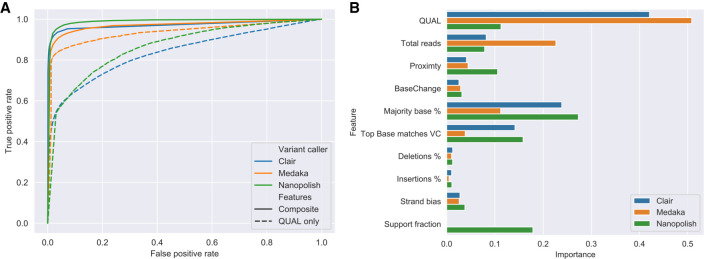
Random forest–based variant filtering using Nanopolish, Medaka, and Clair. (*A*) Receiver operating characteristic (ROC) curve for random forest classifier using different features including Quality (QUAL only, dashed line) and a composite selection of input features (Composite, solid line) for Nanopolish (green), Medaka (orange), and Clair (blue). AUC for each variant caller: Nanopolish 0.86 to 0.98, Medaka 0.93 to 0.97, Clair 0.84 to 0.97, using QUAL and Composite features, respectively. (*B*) Bar chart of feature importance for composite selection of features used to train the classifier.

To improve performance, we trained a random forest classifier to filter the variants using input features from SAMtools and the variant callers (detailed in Methods). Performance was assessed using the 50% of bases in the validation set for each genome across all subsampled depths. This approach improved the area under the curve (AUC) for true SNP identification for Nanopolish from 0.86 using a QUAL threshold alone to 0.98 (Fig. 2A). For Medaka, the AUC improvement was less pronounced, from 0.93 to 0.97. Clair saw the biggest relative improvement from 0.84 to 0.97. The relative importance for each feature varied for each variant caller (Fig. 2B).

#### Impact of depth of coverage

**Figure 3.**
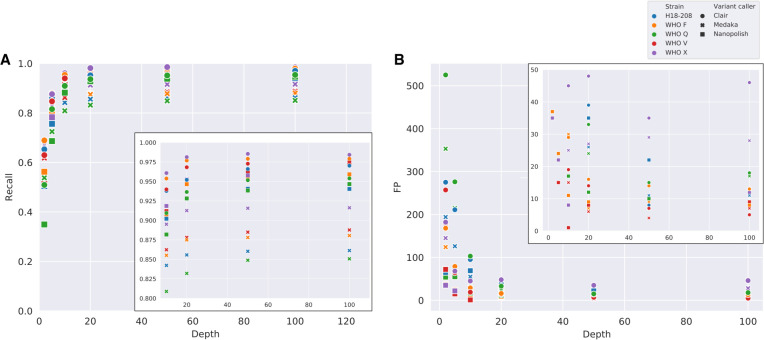
Effect of read coverage depth on SNP calling for each strain and variant caller. (*A*) SNP recall by median depth of coverage. (*B*) False positive SNPs (FP) by median depth of coverage. Color represents different sequences, shapes represent variant callers, circles are Clair, crosses are Medaka, and squares are Nanopolish. *Insets* show *upper* and *lower* regions of the *y*-axis in more detail for *A* and *B*, respectively.

Using our trained classifier, we assessed the impact of depth of coverage on SNP detection, reporting findings across the whole genome. Increasing coverage up to 20× improved SNP detection, for example, using Nanopolish, SNP sensitivity was 0.35–0.56, 0.88–0.92, and 0.93–0.95 at 2×, 10×, and 20× coverage, respectively, across the five genomes (Fig. 3A). Medaka had recall rates ∼5% lower than Nanopolish and Clair. Higher coverage depth also reduced the number of false positive SNPs (Fig. 3B). Nanopolish had the fewest false positives at depths below 20× coverage. At 100× coverage, the numbers of false SNPs per genome ranged from 8 to 13 using Nanopolish (i.e., <1 in 100,000 bases), from 7 to 28 using Medaka, and from 15 to 130 using Clair (Fig. 3), with recall rates of 93%–95%, 85%–92%, and 94%–98%, respectively.

#### Recall performance in important regions and missing SNP calls

We used Clair for subsequent analyses as it offered similar performance to Nanopolish, without requiring resource intensive access to fast5 files. In common with all variant callers tested, Clair missed SNPs (1.5%–3%) such that they were not available at the filtering step. If these errors occur systemically, they do not affect comparisons between genomes; however, if they occur randomly, they can lead to genomes appearing falsely more similar or different.

Missed SNPs were associated with divergence from the reference genome, such that missed SNPs were more closely located to other SNPs (Supplemental Fig. S2A). There was no increase in SNP heterozygosity in genes shared between *Neisseria* species (Supplemental Fig. S2A), suggesting potential contamination from commensal bacteria is being removed by centrifuge in these samples. For antimicrobial resistance prediction, we only called variants on chromosomal genes with low expected diversity and selected the closest reference genes for diverse targets, for example, *penA*. Missed SNPs were not seen within *gyrA*, *porB*, *mtrR*, *parC*, or *ponA* at coverage depths above 10× (Supplemental Fig. S3).

### Antimicrobial resistance determinant identification in conserved genes

Antimicrobial resistance determinants were reliably identified by all three variant callers with only a handful of exceptions. All four copies of the 23S rRNA gene were identified separately using long Nanopore reads. WHO V and WHO Q contain four copies of the A2059G mutation conferring high-level azithromycin resistance. All four mutations were identified at 5×, 10×, or 20× coverage using Clair, Nanopolish, or Medaka, respectively (Supplemental Table S3). Mutations conferring substitutions at positions 91 and 95 in *gyrA* and at positions 86–88 in *parC* confer ciprofloxacin resistance. These amino acids were correctly identified in *gyrA* for all genomes at ≥10× coverage with Clair and Nanopolish, but Medaka failed to detect 95N in WHO X at any depth. Expected results were obtained for *parC* for all variant callers even at 2× depth (Supplemental Table S4). Similarly, *ponA* and *rpsJ* mutations (associated with penicillin and tetracycline resistance, respectively) were identified at all depths with all variant callers.

Two different types of mutations were examined for the *mtrR* gene, the G45D substitution, and promoter variants, which are associated with resistance to azithromycin, ceftriaxone, penicillin, and tetracycline. The amino acid at position 45 was correctly called for all genomes at all depths and with all variant callers, except at 2× coverage for WHO Q with Medaka (Supplemental Table S4). A single-base deletion within the promoter, present within all genomes studied except WHO F, was also detected. Because the reference sequence contained the deletion, it was expected to be detected as an insertion in WHO F. This insertion was only detected by Nanopolish with 100× coverage. Medaka and Clair detected the insertion at all depths, but also incorrectly identified the insertion in WHO X at ≤5× coverage (Supplemental Table S5). As indels were not part of our SNP filtering, we developed a heuristic filter for the insertion: 40% or more reads containing an inserted adenosine, with a coverage depth above 5×, suggested wild-type genotype (Supplemental Fig. S4).

#### penA characterization using whole-genome and local de novo assemblies

The *penA* gene, associated with penicillin and ceftriaxone resistance, is a chromosomal antimicrobial resistance determinant with relatively high nucleotide sequence variation within *N. gonorrhoeae* species arising from recombination events. We identified it using whole-genome and local de novo assemblies followed by mapping the closest known allele.

The required average coverage depth to generate contigs containing the *penA* gene was variable between strains (Supplemental Table S6): H18-208, WHO Q, WHO X, WHO V consistently providing the correct allele with depths of ≥10×. WHO F required 50× coverage for the whole-genome assembly (WGA) method to recall the allele. The local assembly approach worked for all strains from 10× coverage and higher, and it showed better sensitivity at lower read coverage, but did not provide as much genomic context.

#### Detection of plasmid-mediated resistance determinants

Plasmid-carried *tetM* and *blaTEM-1* confer tetracycline and penicillin resistance, respectively. Reads containing *tetM* or *blaTEM-1* sequence were extracted and assembled. To determine if the plasmids were consistent with those in *N. gonorrhoeae* rather than other contaminating species present, we analyzed the gene and flanking plasmid sequence. To reliably confirm the presence of these genes, contigs containing *blaTEM-1* or *tetM* needed to share >60% sequence proportion matching a known carrier plasmid (Supplemental Fig. S5) with >95% sequence identity. Using this heuristic threshold, it was possible to correctly determine that WHO Q and WHO V contained *tetM* and *blaTEM-1,* respectively.

### Longer reads improve metagenomic species disentanglement

To avoid erroneous results arising from DNA from other species, only reads classified as *Neisseria gonorrhoeae* to the species level were used for analysis. By limiting the analysis to only this subset of reads, there is a risk of missing regions of the genome by filtering reads that assign to a lower taxon (Nasko et al. 2018). We therefore tested the expected proportion of the *N. gonorrhoeae* genome that would be classified to the species level by simulation (Supplemental Fig. S6). In contrast to other species, *N. gonorrhoeae* could reliably be identified to the species level with read lengths of a few hundred base pairs. The mean read length from our sequencing was between 2 and 4 kb (Street et al. 2020), which enabled a high proportion of *N. gonorrhoeae* sequence to be assigned to the species level. Furthermore, given Centrifuge's ability to distinguish between closely related *Neisseria* species (Supplemental Fig. S6), we expect this process to be applicable to other samples such as nasopharyngeal swabs that often contain commensal *Neisseria* species.

### Further filtering to remove false SNP calls

When using SNP data to reconstruct transmission events, false SNPs can lead to transmission being incorrectly excluded or deemed unlikely. Similarly, missed SNPs occurring at random, in which the consensus sequence is wrongly set to be wild type, can increase measured genetic distance between two similar strains. In contrast, the expected sequence difference at filtered sites, where the base is unknown, can be adjusted for in proportion to the percentage of the genome filtered and variation in the known genome. Therefore, for transmission studies, a strategy of favoring removing false positive and false negative SNPs over recall is preferred. To achieve this, the SNP classifications were further filtered by masking nucleotide classifications to N if the proportion of bases at a given position supporting the classification was less than 0.8. This value was chosen as the proportion of true positive SNPs with support less than 0.8 is relatively low, but this threshold is sufficiently high to avoid most false negative calls (Supplemental Fig. S7).

By using this final filter with Clair base-called data at 100× coverage, the number of false positive SNPs was reduced from 15–130 to 9–35 across the five genomes analyzed (Table 2). The number of false negative SNPs also fell from 49–249 to 4–19. Overall this resulted in false SNP rates (false negative + false positive SNPs) falling from 66–428 to 15–45, with a reduction in recall from 0.93–0.99 to 0.76–0.94, which is likely to still remain acceptable for most transmission studies.

**Table 2.**
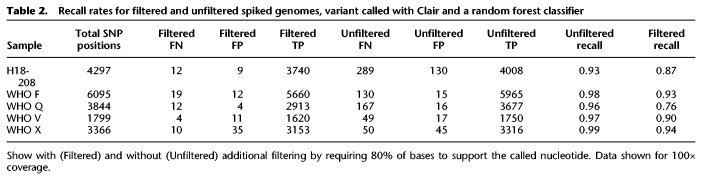
Recall rates for filtered and unfiltered spiked genomes, variant called with Clair and a random forest classifier

### Application of the workflow on clinical samples

We analyzed previously generated Nanopore metagenomic sequencing data from 10 urine samples from men with urethral gonorrhea. We compared findings with our workflow to Illumina data obtained as part of this study from sequencing isolates from the same infections. By Nanopore sequencing, ≥92.8% coverage of an *N. gonorrhoeae* reference genome was achieved in all samples, with ≥93.8% coverage breath at ≥10-fold depth in seven samples.

All resistance gene SNPs were correctly identified in the metagenomic clinical samples (Table 3). Using the heuristic method, the *mtrR* promoter deletion was correctly detected in samples 202, 250, 301, and 314, and the wild-type sequencing in samples 271, 294, and 315. However, sample 303 was incorrectly identified, with only 11× mean genome coverage depth and 8× coverage over the *mtrR* gene suggesting a lack of sequencing depth to accurately call the position (Table 3). The *penA* allele was correctly identified in nine of the 10 clinical samples (Supplemental Table S7). All clinical metagenomic samples identified corresponded with Illumina sequenced cultures at 100% identity according to BLASTN results. Sample 303 produced insufficient data to detect the *penA* gene. It was also possible to determine that samples 206, 271, 294, and 304 contained the *tetM* gene on the pEP5050 plasmid, and samples 294 and 303 contained the *blaTEM-1* gene on the pEM1 plasmid (Supplemental Fig. S8).

**Table 3.**
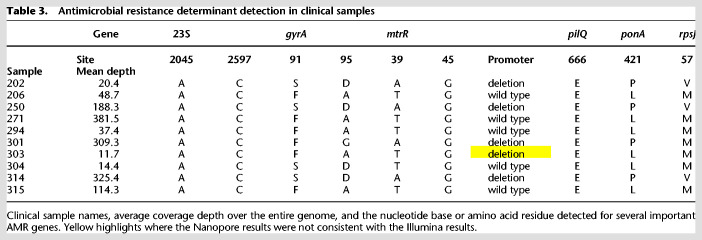
Antimicrobial resistance determinant detection in clinical samples

**Figure 4.**
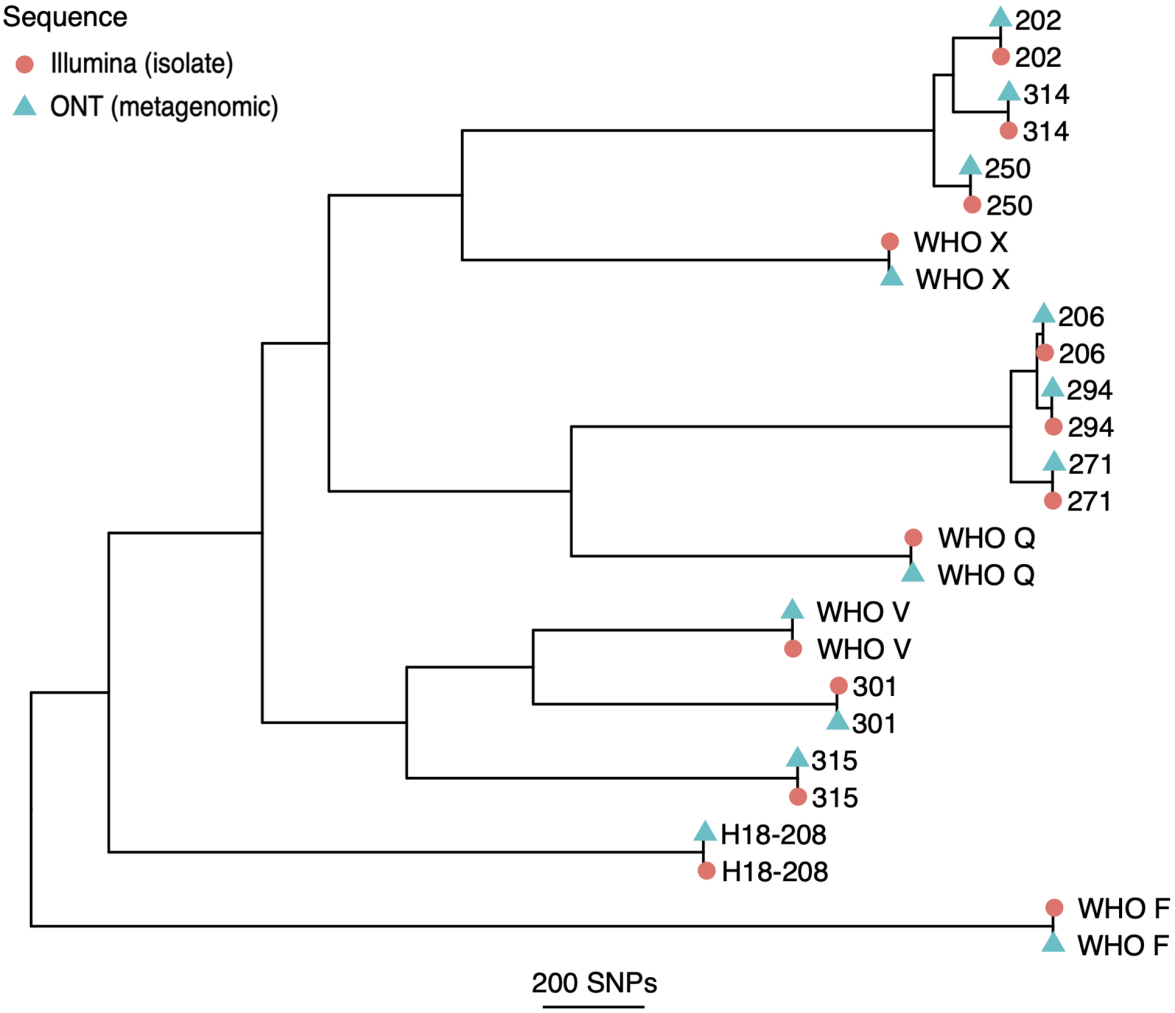
Recombination-corrected maximum likelihood tree of metagenomic Nanopore and paired Illumina isolate sequences. All Nanopore consensus sequences were generated from metagenomic sequencing with the exception of H18-208 and WHO Q, which were sequenced from isolates.

By producing a Nanopore consensus sequence with only high probability SNPs added, and sites with <80% support set to N (i.e., unknown), conventional tree building methods can be used. This approach showed comparable findings between cultured isolates sequenced with Illumina and clinical metagenomic samples sequenced with Nanopore (Fig. 4). Samples 303 and 304 provided insufficient data to generate complete consensus sequences (only 53% and 56% of the reference genome length was identified). For the remaining eight clinical samples and five method development sequences, the median (IQR) [range] genetic distance between the Illumina and Nanopore sequences from the same infection was 5 (3–6) [1–10] SNPs, which is close enough to make transmission studies possible using metagenomic data alone.

### Time to results

Speed of determining antimicrobial resistance is an important goal of clinical metagenomics. Laboratory work before sequencing starts takes 6–7 h. This includes DNA extraction of 2–3 h and sequencing library preparation of 4.5 h (which includes a 3.5-h PCR step). From the start of a sequencing run it took a range of times for the clinical samples to reach 20× coverage required for confident analysis (Supplemental Fig. S9). Four samples, 301, 314, 271, and 250, took <1 h. Sample 315 took <2 h. Samples 294 and 206 took 5 h and 9 h, respectively. Samples 202 and 304 passed 10× coverage after 6 h, and sample 303 never reach 10× coverage.

## Discussion

We show an approach that allows Nanopore sequencing data to be used to reconstruct accurate consensus bacterial genomes. This can be done without accompanying Illumina short-read data and can be applied to metagenomic sequencing data. We show the reconstructed genomes allow accurate resistance detection and transmission inferences to be made in *N. gonorrhoeae*, including using samples obtained from clinical infections.

We evaluated three variant callers, Nanopolish, Medeka, and Clair, against Illumina variant calling from sequenced cultures. After filtering variant calls with a trained random forest classifier, we found that Clair performed better than Nanopolish and Medaka, identifying 94%–98% of SNPs present in Illumina sequences at 100× coverage, compared to 93%–95% and 85%–92%, respectively. Initially, Clair had the highest number of false positive SNPs per genome (15–130, compared to 8–13 and 7–28, respectively). By using further filtering, requiring the proportion of reads supporting any call to be ≥0.8, the number of false positive SNPs could be reduced using Clair to 4–35/genome, albeit with a reduction in SNP detection to 76%–94%. This filtering and masking approach also reduced the number of false negative SNPs from 49–289/genome to 4–19/genome, that would otherwise increase genetic distance during phylogenetic inference. For variant detection in resistance genes specifically, Clair was able to detect all the important SNPs with a coverage of 10× and above, whereas Medaka missed an important SNP in the WHO X strain.

Medaka (v0.10) is still an early release experimental research tool that is focused more on diploid variant calling and haplotype phasing rather than the application tested here. Medaka and Clair have the advantage of not requiring the fast5 files, which have a huge storage and computational requirement. One limitation for neural network–based variant callers, including Clair and Medaka, is understanding the decisions made to call positions. The threshold analysis workflow written here has been designed to drop in different variant calling components to allow for testing new variant calling options in future.

Because our Illumina data truth set pipeline only produced SNP calls, our current variant call filtering was limited to SNPs. Indels were not considered except for the *mtrR* promoter region where a bespoke heuristic method was used. Therefore, further work on the Illumina sequences will be needed to provide a truth set for indel data to allow the development of robust indel calling from Nanopore data, which may also improve with future Nanopore pore technology.

By subsampling reads to produce artificially reduced coverage depths, we have determined the required depth needed to accurately call variants from Nanopore data: 10× fold coverage is sufficient to define resistance determinants with minimal increase in recall above 20× fold coverage.

We were successfully able to detect relevant *N. gonorrhoeae* antimicrobial resistance determinants conferring resistance to clinically important antibiotics across all samples tested with a coverage depth above 10×. Most variants could be detected from appropriately filtered variant calls from mapped data, and *penA* allele determination could be achieved using a combined assembly and mapping approach. The WGA approach provided more genomic context around the *penA* allele that could guarantee the allele was from *Neisseria gonorrhoeae* and not a contaminating commensal, whereas local gene assembly (LGA) performed better at lower read depths. Ra was chosen as the assembler for WGA as WTDBG2 (redbean) produced some misassembly that prevented remapping of reads to the *penA* locus (Supplemental Fig. S10). However, Ra failed to produce contigs for most of the attempts when used for the LGA. It was possible to recover the four 23S rRNA loci separately from each sample containing the expected A2059G mutation. This was not possible using short-read Illumina sequencing. The Nanopore read length allowed us to span the entire gene with enough genomic context to confidently map to each locus independently. We predict the Centrifuge and mapping strategy will distinguish between closely related *Neisseria* species commonly found in nasal pharyngeal samples. Further sequencing of samples from these sites would be required to empirically show this strategy works.

Our final consensus sequence comparison yielded a median of five SNPs between Illumina and Nanopore sequences. Although this does not match the reproducibility seen in Illumina sequencing of isolates (De Silva et al. 2016), it is close enough to judge whether infections are part of specific transmission clusters (De Silva et al. 2016), even if precise reconstruction of individual transmission events may remain challenging with Nanopore data alone. A limitation of this study was only using the NCCP11945 reference genome. Using a reference genome more closely related to a cluster of genomes may reduce the false positive variant call rate to enable transmission analysis, as seen with Illumina data (Bush et al. 2020). However, this is beyond the scope of this analysis and will be attempted in future work with samples from the same clusters.

The bioinformatic workflow takes ∼30 min to run. However, the biggest bottlenecks are species classification, read binning, and mapping. These could be optimized further, or run in real time as shown previously (Sanderson et al. 2018), which performs these steps as read files are produced and continuously combines the output into a single sorted BAM file.

The current generation of ONT flow cells used in this analysis is R9.4.1. However, new pores such as R10 are currently in development and may offer increased accuracy. The validation part of this workflow should be run on new sequences generated by future pores to set new threshold values and filtering models that are appropriate to these new pore error profiles.

The approaches we have developed provide a mechanism for determining antimicrobial resistance and undertaking transmission tracking using clinical samples. This, taken together with recent advances in optimizing DNA extraction for metagenomic Nanopore sequencing of *N. gonorrhoeae* direct from urine samples (Street et al. 2020), now provides an opportunity to test the performance of Nanopore sequencing as a clinical diagnostic in *N. gonorrhoeae* infection. Furthermore, this approach may have wide applicability across a range of bacterial pathogens, not just *N. gonorrhoeae,* where bacterial genomes can be successfully disentangled from metagenomic samples with modest sequencing read lengths. Evaluations in clinical data sets will allow the potential utility of our approaches to be further investigated and potentially provide new diagnostics to guide patient and public health management of gonorrhea.

## Methods

We developed an optimized workflow to deliver several outputs from metagenomic sequence data containing *N. gonorrhoeae*: (1) classification of sequence reads by species of origin to allow the presence/absence of *N. gonorrhoeae* to be determined, (2) identification of *N. gonorrhoeae* antimicrobial resistance determinants, and (3) a consensus whole-genome sequence to facilitate comparisons between genomes for tracking transmission.

### Data sources

To develop and test the performance of our workflow, we used ONT data generated in a previous study (Street et al. 2020) from metagenomic sequencing of *N. gonorrhoeae* nucleic acid amplification test (NAAT)-negative urine samples spiked with varying concentrations of three WHO *N. gonorrhoeae* reference strains: WHO F, WHO V, and WHO X. Sequencing was described previously (Street et al. 2020). Briefly, samples were sequenced on ﻿FLO-MIN106D (v.R9.4.1) flow cells ﻿using the Rapid PCR barcoding kit (SQK-RPB004) (ONT), with modifications to the manufacturer's protocol as described previously (Charalampous et al. 2019). Additional data from ONT sequences of isolates WHO Q (Eyre et al. 2018; Jennison et al. 2019) and H18-208 (Eyre et al. 2019) were also used, using the same flow cells. Details of sequences and accession numbers are given in Table 1. ONT data were compared with Illumina data available for the reference strains and clinical isolates, which were used as a gold standard together with published descriptions of the variants present (Unemo et al. 2016; Eyre et al. 2018, 2019). Illumina data were processed as described previously (De Silva et al. 2016; Eyre et al. 2017).

In addition, we also tested our final algorithm on 10 Nanopore metagenomic sequences from *N. gonorrhoeae* positive urine samples obtained from men with symptomatic urethral gonorrhea, described previously (Street et al. 2020). Cultured isolates from the same infections were sequenced with an Illumina MiniSeq, following the manufacturer's instructions, to allow for comparisons.

### Workflow

Our end-to-end workflow is written in Nextflow's domain specific language (Di Tommaso et al. 2017) and consists of various open source programs and databases (Supplemental Fig. S11). A second workflow was used to determine the thresholds needed to filter SNPs based on input sequences, truth sequences, and the variant caller used. Both workflows can also be found within a GitLab repository (https://GitLab.com/ModernisingMedicalMicrobiology/ngonpipe).

### Base calling

Raw Nanopore reads were base called with Guppy version 3.1.5 + 781ed57 using the high accuracy HAC models (dna_r9.4.1_450bps_ hac.cfg, template_r9.4.1_450bps_hac.jsn). Runs had single barcodes per flow cell and so were not demultiplexed.

### Read classification with centrifuge and read binning

Taxonomic classification of base-called Nanopore reads was performed using Centrifuge version 1.0.4-beta (Kim et al. 2016), with a database built from NCBI RefSeq genomes including bacteria and virus genomes deposited as of August 10, 2018, as well as the Human hg38 reference genome. Centrifuge was run with a minimum hit length of 16 (‐‐min-hitlen 16) and reporting a single distinct primary assignment for each read (-k 1). Reads that were classified as, or were a strain of, *N. gonorrhoeae* were collected in a separate FASTQ file using a custom Python script (bin_reads.py) available within the GitLab repository.

### Genome alignment

To reduce errors arising from reads from other species mapping to similar genes in the *N. gonorrhoeae* genome, as observed in other metagenomic samples, for example, with *Mycobacterium tuberculosis* (Wyllie et al. 2018), only reads classified as *N. gonorrhoeae* were aligned. *N. gonorrhoeae*reads were mapped to the NCCP11945 *N. gonorrhoeae* reference genome (accession NC_011035.1) using minimap2 version 2.17-r941 (Li 2018) using settings for Nanopore data (-ax map-ont). Aligned reads were filtered to remove alignments with a map quality score less than 50 and sorted and indexed using SAMtools version 1.9 (Li et al. 2009).

### Subsampling genome depth

To understand the effect of read depth on variant calling accuracy, aligned BAM files for each of the five isolates were subsampled. A custom wrapper script (subSampleBam.py, in the GitLab repository) for “SAMtools view” (Li et al. 2009) was used to target 2, 5, 10, 20, 50, and 100× average coverage depths.

### Variant calling

Variants were called from the aligned Nanopore reads to either the full genome or, for variable genes, after remapping to the closest available resistance gene allele from the NG-STAR database (https://ngstar.canada.ca). Several variant callers were tested. Nanopolish version 0.11.1 (Simpson et al. 2017) was used with the methylation aware options (‐‐methylation-aware dcm,dam), ‐‐fix-homopolymers, and ploidy set to 1 (‐‐ploidy 1). Medaka version v0.10.0 (https://github.com/nanoporetech/medaka) was used with the consensus and variant subcommands. Clair callVarBam (git commit 54c7dd4) (Luo et al. 2020) was used with default ONT settings. Additional information was acquired from pysamstats version 1.1.2 (https://github.com/alimanfoo/pysamstats, pysam 0.15.2) using the variation strand (-t variation_ strand) option.

Variants identified by the variant callers were filtered based on metrics generated by pysamstats together with Nanopolish, Medaka, or Clair. Filtering was undertaken using a random forest classifier, using the scikit-learn package (Pedregosa 2011), by comparing Nanopore variant caller outputs and “truth” data from Illumina sequencing of the same isolate. Because the purpose of the classifier was to filter potential variants identified by the variant caller, only these sites were used for training. However, summaries of the performance of the classifier at the whole-genome level are provided in the results. We defined true positive (TP) SNPs as those that were called and passed by both methods, false positive (FP) Nanopore SNPs that were not found with Illumina sequencing, and true negative (TN) sites were called as wild type by both methods. Sites could be falsely negative (FN) by Nanopore when an Illumina SNP was either missed by the variant caller initially or filtered out incorrectly by the random forest classifier.

To train and test the classifier, we used the Nanopore and Illumina sequence for each of the five isolates. To include read depth as a component of the SNP classification, the five genome strains were subsampled to six target depths of 2, 5, 10, 20, 50, and 100× coverage. All sites from each of the 30 subsampled genomes were randomly divided into a 50% training and 50% validation set. Default hyperparameter values were used. Reported performance metrics include sensitivity or recall, Recall = *TP*/(*TP* + *FN*) and precision (or positive predictive value for a variant call), Precision = *TP*/(*TP* + *FP*).

We considered the following additional metrics obtained using Nanopolish and pysam as input features for the classifier: Variant quality (QUAL), Nanopolish support fraction (Support fraction), total number of reads aligned to each position (Total reads), proximity to the nearest variant in base pairs (proximity), the combination of reference and variant base (baseChange), the proportion of bases the same as the majority base (majority base %), concordance between dominant base and the variant reported (Top base matches variant caller), the proportion of reads in each direction (strand bias), and proportion of reads that are indels (deletions %, insertions %). This was repeated for Medaka and Clair with the exception of the Support fraction metric that is specific to Nanopolish.

### Heterozygosity in shared genes

Prokka v1.14.6 (Seemann 2014) and Roary v3.13.0 (Page et al. 2015) were used to identify shared genes between several different species including *N. gonorrhoeae* (NC_002946.2), *N. meningitidis* (NC_003112.2), *N. lactamica* (NC_014752.1), *N. elongata* (NZ_ CP007726.1), *N. mucosa* (NZ_CP020452.2), *N. subflava* (NZ_ CP031251.1), *N. cinerea* (NZ_LS483369.1), *N. weaveri* (NZ_LT571436.1), and *N. zoodegmatis* (NZ_LT906434.1). Genes that were shared by two or more species with >95% identity were described as shared genes. SNPs classified by Clair and filtered by the trained random forest were used to visualize the difference in supporting bases (additional data from pysamstats) between different samples and shared gene status.

### Indel detection within the *mtrR* promoter

Indels were detected at specific positions within the BAM file using a bespoke Python script (indel_class.py, available in the GitLab repository) that uses pysam (https://github.com/pysam-developers/pysam) to count the proportion of inserted reads at a position.

### Whole-genome assembly (WGA)

Binned reads were filtered for length and quality using Filtlong commit 13504b7 (https://github.com/rrwick/Filtlong) for a minimum length of 1000 bp (‐‐min_length 1000), keeping up to 90% of bases (‐‐keep_percent 90) and using a target bases value of 500 mega bases (‐‐target_bases 500000000) as determined in previous work on long-read assembly (De Maio et al. 2019). Filtered reads were assembled into contigs using Ra commit 07364a1 (https://github.com/lbcb-sci/ra) using the -x ont parameters.

### Local gene assembly (LGA) and remapping for *penA* characterization

For highly variable genes, that is, *penA*, mapping to a single reference sequence was not possible given the diversity present. Therefore, reads containing genes of interest were identified and isolated using minimap2 and the bin_reads.py script. These local reads were subsequently assembled using wtdbg2 version 2.3 (Ruan and Li 2020) with a longest subread of 3 kb (-L 3000), that is, the default setting at the time the workflow was developed. A database of available alleles for *penA* was created using the alleles available within the NG-STAR database (https://ngstar.canada.ca). The closest matched allele for each gene was determined using BLASTN (Altschul et al. 1990) to search the LGA/WGA contigs. The closest match was chosen as having >95% subject coverage and the highest bitscore. The closest matched allele was then used as a reference to realign binned reads against, using the same mapping and variant calling methods described above.

### *Neisseria gonorrhoeae* antibiotic resistance determinant identification

Following the data processing outlined above, the remaining antimicrobial resistance determinants were identified similarly to our previous approach (Eyre et al. 2017) developed for short-read sequencing of isolates. Variants in the following genes in the NG-STAR scheme were sought: *penA*, *mtrR*, *porB*, *ponA*, *gyrA*, *parC*, 23S rRNA, as well as *rpsJ* mutations and *tet* family genes conferring resistance to tetracycline. Amino acid changes were identified using variant calls in VCF format converted to consensus DNA sequences and then translated. Mutations and variants in promoter sequences were identified from the consensus DNA sequences.

For *penA,* exact matches with one of the alleles in the NG-STAR database were sought (because all isolates/references sequenced were already in the database), but variation from these could also be detected.

To identify mutations in each of the four copies of the 23S rRNA genes associated with macrolide resistance, the four 23S rRNA loci were independently examined for depth of coverage and base changes. This is in contrast to previous approaches using short-read data in which the different loci had to be analyzed together by mapping to a single copy of the gene (Eyre et al. 2017).

Antimicrobial resistance conferred by the presence of a specific accessory gene, for example, plasmid-associated *tetM/blaTEM-1*, was identified using an assembly strategy. Reads were identified using minimap2 overlaps (-x ava-ont) of all the base-called reads against a database of accessory gene sequences and assembled with wtdbg2. The resulting contigs were analyzed for *tetM/blaTEM-1* sequence and known carrier plasmids for *Neisseria gonorrhoea*e using BLASTN searches of the same database including pEP5289 (GU479464), pEP5233 (GU479465), pEP5050 (GU479466) for *tetM* (Pachulec and van der Does 2010) and pEM1 (HM756641.1), pGF1 (U20421), pJD5 (U20375) and pJD7 (U20419) for *blaTEM-1* (Müller et al. 2011).

### Phylogenetic inference

We compared phylogenetic inferences using Nanopore and Illumina data using whole-genome consensus sequences produced after filtering. To reduce the number of false positive and false negative Nanopore SNP calls, we also tested additionally masking positions (i.e., setting the base to N) where the proportion of reads supporting the called base was less than a given threshold, for example, 0.8. Maximum likelihood phylogenetic trees were constructed with IQ-TREE (v1.6.1) (Chernomor et al. 2016) and branch lengths readjusted to account for recombination with ClonalFrameML (v1.11-1) (Didelot and Wilson 2015) using default settings. The workflow used is provided within the Nextflow workflow and is based on runlistcompare (https://github.com/davideyre/runListCompare).

## Data access

The Nanopore data generated in this study, including fast5 files, have been submitted to the NCBI BioProject database (https://www.ncbi.nlm.nih.gov/bioproject/) under accession numbers PRJEB35173 and PRJEB26560. Illumina sequenced culture isolates are available under accession number PRJNA603903. Data analysis workflow is available as a Git repository (https://gitlab.com/ModernisingMedicalMicrobiology/ngonpipe) and as Supplemental Code.

## Competing interest statement

D.W.E. has received lecture fees and expenses from Gilead unrelated to the current study. The other authors declare no competing interests.

## Supplementary Material

Supplemental Material
