# Supplementary material for: High precision Neisseria gonorrhoeae variant and antimicrobial resistance calling from metagenomic Nanopore sequencing
Source: Genome Res. 2020 Sep;30(9):1354–63. doi: 10.1101/gr.262865.120 (PMC7545138; doi:10.1101/gr.262865.120)
Supplement: Supplemental Material [file supp_30_9_1354__index.html]

High precision Neisseria gonorrhoeae variant and antimicrobial resistance calling from metagenomic Nanopore sequencing — Supplemental Material 

# High precision *Neisseria gonorrhoeae* variant and antimicrobial resistance calling from metagenomic Nanopore sequencing

## Supplemental Material

- Supplemental\_Code.tar.zip
- Supplemental\_Material.docx
