# Supplementary material for: High precision Neisseria gonorrhoeae variant and antimicrobial resistance calling from metagenomic Nanopore sequencing
Source: Genome Res. 2020 Sep;30(9):1354–63. doi: 10.1101/gr.262865.120 (PMC7545138; doi:10.1101/gr.262865.120)
Supplement: Supplemental Material [file supp_gr.262865.120_Supplemental_Material.docx]

## Supplemental Figure legends

Supplemental figure 1**. Sequence coverage depth and breadth in original and subsampled data.** (A) Median depth achieved from subsampling bam files and original unsampled bam files (Full). (B) Coverage breadth at each target depth.

Supplemental figure 2**. Box and whisker plots for found genes by variant callers and positions within genomes.** (A) box and whisker plots of detected and missed SNPs and proximity to closest genuine SNP using each variant caller. Box and whisker plots of detected SNPs (Clair) and % of reads concordant with the majority base, split between SNPs in genes shared with other Neisseria species (True, orange) or N. gonorrhoea species genes or intergenic regions (False, blue).

Supplemental figure 3**. SNP detection in N. gonorrhoeae genes associated with antimicrobial resistance**. Bar plots showing number of positions with different SNP types (columns) detected by Clair in important genes (rows). From left to right: gyrA, porB, mtrR, parC, ponA. From top to bottom: True positives (TP), False positives (FP), False negatives (FN), Missed SNPs, no reads covering gene. All plots share a y-axis axis of number of positions classified and x-axis of target depth of coverage. Colours represent each sequence used. The 23S gene was masked from Illumina mapped reads as the read lengths were too short to distinguish different loci and are therefore not compared here.

Supplemental figure 4**. mtrR promoter deletion of adenosine base.** The reference genome used contains the deletion, and the true wild-type is represented by an insertion. Percentage of reads with an inserted base at position 1332810 in reference NC_011035.1.

Supplemental figure 5. **detection of plasmid-borne resistance genes.** Proportion of the query sequence (assembled contig) matching a known carrier plasmid (y-axis) over validation of the sample containing tetM or blaTEM-1 (x-axis), colours represent samples (A). Percentage identity between the matching contigs and carrier plasmid (y-axis) over proportion of the query sequence matching a known carrier plasmid (x-axis), colour represents validation of containing tetM or blaTEM-1 (B).

Supplemental figure 6**. Taxonomic assignment by read length**. Simulated raw reads for several Neisseria species including gonorrhoeae. Staphylococcus aureus and Mycobacterium tuberculosis are included for comparison. Colours represent each species, line styles represent whether part of the Neisseria genus.

Supplemental figure 7**. Filtering SNP calls based on support rate**. Proportion of SNPs that are true positives classified by weak support (True equates to less than the support rate, orange or False equates to more that the support rate, blue) over support rate (A). The number of positions that are False Negatives (B) or False Positives (C) over support rate and classified by weak support (same as A).

Supplemental figure 8**. Plasmid-borne resistance gene detection in clinical samples.** Proportion of the query sequence (assembled contig) matching a known carrier plasmid (y-axis) by presence of tetM or blaTEM-1 in the sample (x-axis), colours represent samples (A). Percentage identity between the matching contigs and carrier plasmid (y-axis) over proportion of the query sequence matching a known carrier plasmid (x-axis), colour represents validation of containing tetM or blaTEM-1 (B).

Supplemental figure 9. **Estimated genome coverage over time from run start.** Cumulative estimated genome coverage over time for each of the ten clinical samples sequenced. Coverage is estimated by dividing the number of bases sequenced classified as N. gonorrhoea by the length of the reference genome. Horizontal line shows 20x coverage, the amount required for a confident detection of variants.

Supplemental figure 10 **Example of penA miss-assembly.** Nearly correct penA RA assembly from WHO F reads at 20x subsampled coverage depth shows 1746 bp alignment with allele 15.001 with 99% identity and 3 gaps (A). Truncated penA wtdbg2 assembly from WHO F reads at 20x subsampled coverage depth shows 384 bp length alignment with allele 15.005 with 91% identity and 24 bp gaps (B).

Supplemental figure 11**. Basic workflow schematic.** For antimicrobial resistance prediction mtrR, porB, ponA, gryA, parC, 23S rRNA are identified from the consensus sequence obtained from mapping to the default reference, penA uses a custom reference - see right-hand branch of schematic. Genes present on plasmids (tetM, blaTEM) are identified by mapping to a collection of accessory genes.

## Supplemental Tables

| Strain | Illumina bases covered | Reference Chromosome length | Missed (N) sites | Illumina SNPs | Invariant sites |
| --- | --- | --- | --- | --- | --- |
| H18-208 | 1,992,319 | 2,232,025 | 239,706 | 4,303 | 1,988,016 |
| WHO Q | 1,962,756 | 2,232,025 | 269,269 | 3,844 | 1,958,912 |
| WHO F | 1,991,234 | 2,232,025 | 240,791 | 6,174 | 1,985,060 |
| WHO V | 2,016,466 | 2,232,025 | 215,559 | 1,801 | 2,014,665 |
| WHO X | 1,955,978 | 2,232,025 | 276,047 | 3,366 | 1,952,612 |

Supplemental table 1**. “Gold standard” variation present from Illumina sequencing.** Number of variants, invariant sites, and uncalled sites found within each sample from Illumina sequencing.

| Metric | FP | | | | | | | | | | | | | | | | | |
| --- | --- | --- | --- | --- | --- | --- | --- | --- | --- | --- | --- | --- | --- | --- | --- | --- | --- | --- |
| **Variant caller** | **Clair** | | | | | | **Medaka** | | | | | | **Nanopolish** | | | | | |
| **Depth** | **2** | **5** | **10** | **20** | **50** | **100** | **2** | **5** | **10** | **20** | **50** | **100** | **2** | **5** | **10** | **20** | **50** | **100** |
| **Strain** |  |  |  |  |  |  |  |  |  |  |  |  |  |  |  |  |  |  |
| **H18-208** | 290 | 1007 | 1760 | 2294 | 2378 | 1839 | 161 | 147 | 63 | 40 | 11 | 18 | 1362 | 1792 | 1893 | 1992 | 1896 | 1870 |
| **WHO F** | 1420 | 2408 | 1855 | 1066 | 543 | 319 | 98 | 88 | 47 | 26 | 26 | 29 | 505 | 356 | 163 | 77 | 48 | 47 |
| **WHO Q** | 225 | 1244 | 2226 | 2949 | 2948 | 2327 | 271 | 210 | 90 | 30 | 20 | 26 | 1355 | 1809 | 1542 | 1478 | 1410 | 1263 |
| **WHO V** | 1431 | 2640 | 1741 | 581 | 116 | 39 | 127 | 71 | 17 | 12 | 12 | 8 | 911 | 361 | 97 | 40 | 48 | 39 |
| **WHO X** | 1360 | 2387 | 1447 | 434 | 81 | 55 | 105 | 85 | 54 | 58 | 63 | 67 | 340 | 297 | 101 | 73 | 42 | 32 |
| **Metric** | **Precision** | | | | | | | | | | | | | | | | | |
| **Variant caller** | **Clair** | | | | | | **Medaka** | | | | | | **Nanopolish** | | | | | |
| **Depth** | **2** | **5** | **10** | **20** | **50** | **100** | **2** | **5** | **10** | **20** | **50** | **100** | **2** | **5** | **10** | **20** | **50** | **100** |
| **Strain** |  |  |  |  |  |  |  |  |  |  |  |  |  |  |  |  |  |  |
| **H18-208** | 0.052 | 0.310 | 0.583 | 0.614 | 0.618 | 0.679 | 0.890 | 0.949 | 0.981 | 0.989 | 0.997 | 0.995 | 0.568 | 0.636 | 0.672 | 0.668 | 0.681 | 0.684 |
| **WHO F** | 0.053 | 0.367 | 0.652 | 0.822 | 0.911 | 0.946 | 0.955 | 0.977 | 0.990 | 0.995 | 0.995 | 0.994 | 0.837 | 0.926 | 0.970 | 0.987 | 0.992 | 0.992 |
| **WHO Q** | 0.004 | 0.231 | 0.451 | 0.506 | 0.532 | 0.592 | 0.728 | 0.909 | 0.967 | 0.989 | 0.993 | 0.991 | 0.441 | 0.587 | 0.688 | 0.709 | 0.721 | 0.743 |
| **WHO V** | 0.006 | 0.090 | 0.361 | 0.723 | 0.934 | 0.977 | 0.800 | 0.943 | 0.988 | 0.992 | 0.992 | 0.995 | 0.448 | 0.795 | 0.944 | 0.977 | 0.973 | 0.978 |
| **WHO X** | 0.014 | 0.204 | 0.566 | 0.872 | 0.975 | 0.983 | 0.906 | 0.964 | 0.981 | 0.981 | 0.980 | 0.978 | 0.794 | 0.891 | 0.968 | 0.978 | 0.987 | 0.990 |
| **Metric** | **Recall** | | | | | | | | | | | | | | | | | |
| **Variant caller** | **Clair** | | | | | | **Medaka** | | | | | | **Nanopolish** | | | | | |
| **Depth** | **2** | **5** | **10** | **20** | **50** | **100** | **2** | **5** | **10** | **20** | **50** | **100** | **2** | **5** | **10** | **20** | **50** | **100** |
| **Strain** |  |  |  |  |  |  |  |  |  |  |  |  |  |  |  |  |  |  |
| **H18-208** | 0.004 | 0.105 | 0.571 | 0.850 | 0.894 | 0.904 | 0.301 | 0.637 | 0.765 | 0.802 | 0.818 | 0.828 | 0.416 | 0.727 | 0.899 | 0.931 | 0.942 | 0.942 |
| **WHO F** | 0.013 | 0.226 | 0.562 | 0.797 | 0.899 | 0.902 | 0.334 | 0.616 | 0.753 | 0.812 | 0.838 | 0.846 | 0.421 | 0.721 | 0.868 | 0.922 | 0.940 | 0.947 |
| **WHO Q** | 0.000 | 0.097 | 0.476 | 0.785 | 0.872 | 0.879 | 0.189 | 0.543 | 0.676 | 0.728 | 0.756 | 0.772 | 0.279 | 0.669 | 0.883 | 0.937 | 0.946 | 0.952 |
| **WHO V** | 0.005 | 0.145 | 0.545 | 0.844 | 0.911 | 0.921 | 0.282 | 0.650 | 0.779 | 0.827 | 0.842 | 0.861 | 0.410 | 0.777 | 0.910 | 0.952 | 0.962 | 0.973 |
| **WHO X** | 0.006 | 0.182 | 0.561 | 0.878 | 0.938 | 0.934 | 0.302 | 0.671 | 0.833 | 0.877 | 0.897 | 0.901 | 0.389 | 0.724 | 0.910 | 0.951 | 0.959 | 0.960 |

Supplemental table 2**. FP rate, Recall and Precision using a simple QUAL based threshold for filtering variant calls**.

|  |  | Discreet A2059G copies | | |
| --- | --- | --- | --- | --- |
| Strain | Depth | Nanopolish | Medaka | Clair |
| WHO Q | 2 | 2 | 2 | 2 |
|  | 5 | 3 | 3 | **4** |
|  | 10 | **4** | 3 | **4** |
|  | 20 | **4** | **4** | **4** |
|  | 50 | **4** | **4** | **4** |
|  | 100 | **4** | **4** | **4** |
| WHO V | 2 | 0 | 1 | 2 |
|  | 5 | 3 | 2 | **4** |
|  | 10 | **4** | **4** | **4** |
|  | 20 | **4** | **4** | **4** |
|  | 50 | **4** | **4** | **4** |
|  | 100 | **4** | **4** | **4** |

Supplemental table 3**. Discreet A2059G mutations discovered in 23s rRNA variant calls**. Strains without 23s rRNA mutations are not shown. The expected number of A2059G mutations in WHO Q and WHO V is 4, as shown in bold.

|  |  |  |  | Medaka | | | | | | Nanopolish | | | | | | Clair | | | | | |
| --- | --- | --- | --- | --- | --- | --- | --- | --- | --- | --- | --- | --- | --- | --- | --- | --- | --- | --- | --- | --- | --- |
| **gene** | **Strain** | **Pos** | **Exp. Res.** | **2** | **5** | **10** | **20** | **50** | **100** | **2** | **5** | **10** | **20** | **50** | **100** | **2** | **5** | **10** | **20** | **50** | **100** |
| **gyrA** | **H18-208** | **91** | **F** | F | F | F | F | F | F | F | F | F | F | F | F | F | F | F | F | F | F |
|  |  | **95** | **A** | A | A | A | A | A | A | G | A | A | A | A | A | G | A | A | A | A | A |
|  | **WHOF** | **91** | **S** | F | F | S | S | S | S | F | F | S | S | S | S | F | F | S | S | S | S |
|  |  | **95** | **D** | D | D | D | D | D | D | G | D | D | D | D | D | G | G | D | D | D | D |
|  | **WHOQ** | **91** | **F** | F | F | F | F | F | F | F | F | F | F | F | F | F | F | F | F | F | F |
|  |  | **95** | **A** | G | A | A | A | A | A | G | A | A | A | A | A | G | A | A | A | A | A |
|  | **WHOV** | **91** | **F** | F | F | F | F | F | F | F | F | F | F | F | F | F | F | F | F | F | F |
|  |  | **95** | **G** | G | G | G | G | G | G | G | G | G | G | G | G | G | G | G | G | G | G |
|  | **WHOX** | **91** | **F** | F | F | F | F | F | F | F | F | F | F | F | F | F | F | F | F | F | F |
|  |  | **95** | **N** | G | G | G | G | G | G | G | N | N | N | N | N | G | N | N | N | N | N |
| **parC** | **H18-208** | **86** | **D** | D | D | D | D | D | D | D | D | D | D | D | D | D | D | D | D | D | D |
|  |  | **87** | **R** | R | R | R | R | R | R | R | R | R | R | R | R | R | R | R | R | R | R |
|  |  | **88** | **S** | S | S | S | S | S | S | S | S | S | S | S | S | S | S | S | S | S | S |
|  | **WHOF** | **86** | **D** | D | D | D | D | D | D | D | D | D | D | D | D | D | D | D | D | D | D |
|  |  | **87** | **S** | S | S | S | S | S | S | S | S | S | S | S | S | S | S | S | S | S | S |
|  |  | **88** | **S** | S | S | S | S | S | S | S | S | S | S | S | S | S | S | S | S | S | S |
|  | **WHOQ** | **86** | **D** | D | D | D | D | D | D | D | D | D | D | D | D | D | D | D | D | D | D |
|  |  | **87** | **R** | R | R | R | R | R | R | R | R | R | R | R | R | R | R | R | R | R | R |
|  |  | **88** | **S** | S | S | S | S | S | S | S | S | S | S | S | S | S | S | S | S | S | S |
|  | **WHOV** | **86** | **D** | D | D | D | D | D | D | D | D | D | D | D | D | D | D | D | D | D | D |
|  |  | **87** | **R** | R | R | R | R | R | R | R | R | R | R | R | R | R | R | R | R | R | R |
|  |  | **88** | **S** | S | S | S | S | S | S | S | S | S | S | S | S | S | S | S | S | S | S |
|  | **WHOX** | **86** | **D** | D | D | D | D | D | D | D | D | D | D | D | D | D | D | D | D | D | D |
|  |  | **87** | **R** | R | R | R | R | R | R | R | R | R | R | R | R | R | R | R | R | R | R |
|  |  | **88** | **P** | P | P | P | P | P | P | P | P | P | P | P | P | P | P | P | P | P | P |
| **ponA** | **H18-208** | **421** | **P** | P | P | P | P | P | P | P | P | P | P | P | P | P | P | P | P | P | P |
|  | **WHOF** | **421** | **L** | L | L | L | L | L | L | L | L | L | L | L | L | L | L | L | L | L | L |
|  | **WHOQ** | **421** | **P** | P | P | P | P | P | P | P | P | P | P | P | P | P | P | P | P | P | P |
|  | **WHOV** | **421** | **P** | P | P | P | P | P | P | P | P | P | P | P | P | P | P | P | P | P | P |
|  | **WHOX** | **421** | **P** | P | P | P | P | P | P | P | P | P | P | P | P | P | P | P | P | P | P |
| **mtrR** | **H18-208** | **45** | **G** | G | G | G | G | G | G | G | G | G | G | G | G | G | G | G | G | G | G |
|  | **WHOF** | **45** | **G** | G | G | G | G | G | G | G | G | G | G | G | G | G | G | G | G | G | G |
|  | **WHOQ** | **45** | **D** | G | D | D | D | D | D | D | D | D | D | D | D | D | D | D | D | D | D |
|  | **WHOV** | **45** | **G** | G | G | G | G | G | G | G | G | G | G | G | G | G | G | G | G | G | G |
|  | **WHOX** | **45** | **G** | G | G | G | G | G | G | G | G | G | G | G | G | G | G | G | G | G | G |
| **rpsJ** | **H18-208** | **57** | **M** | M | M | M | M | M | M | M | M | M | M | M | M | M | M | M | M | M | M |
|  | **WHOF** | **57** | **V** | V | V | V | V | V | V | V | V | V | V | V | V | V | V | V | V | V | V |
|  | **WHOQ** | **57** | **M** | M | M | M | M | M | M | M | M | M | M | M | M | M | M | M | M | M | M |
|  | **WHOV** | **57** | **M** | M | M | M | M | M | M | M | M | M | M | M | M | M | M | M | M | M | M |
|  | **WHOX** | **57** | **M** | M | M | M | M | M | M | M | M | M | M | M | M | M | M | M | M | M | M |

Supplemental table 4**. Genes and expected mutations for each strain at varying subsampled depths using Nanopolish or medaka for variant calling**. Residues highlighted in yellow are incorrect.

| Strain | Variant caller | 2 | 5 | 10 | 20 | 50 | 100 |
| --- | --- | --- | --- | --- | --- | --- | --- |
| **WHO F** | **Clair** | TA | TA | TA | TA | TA | TA |
|  | **Medaka** | TA | TA | TA | TA | TA | TA |
|  | **Nanopolish** |  |  |  |  |  | TA |
| **WHO X** | **Clair** |  | TA |  |  |  |  |
|  | **Medaka** | TAA | TA |  |  |  |  |

Supplemental table 5**. mtrR promoter sequences**. ALT sequences identified at positions 1332810 in reference NC_011035.1 by each variant caller at different sub sampled depths.

| Strain | Method | 2 | 5 | 10 | 20 | 50 | 100 |
| --- | --- | --- | --- | --- | --- | --- | --- |
| H18-208 | WGA |  |  | 60.001 | 60.001 | 60.001 | 60.001 |
|  | LA |  |  | 60.001 | 60.001 | 60.001 | 60.001 |
| WHO F | WGA |  |  |  |  | 15.001 |  |
|  | LA |  | 15.001 | 15.001 | 15.001 | 15.001 | 15.001 |
| WHO Q | WGA |  |  |  | 60.001 | 60.001 | 60.001 |
|  | LA |  | 60.001 | 60.001 | 60.001 | 60.001 | 60.001 |
| WHO V | WGA |  |  | 5.002 | 5.002 | 5.002 | 5.002 |
|  | LA |  | 5.002 | 5.002 | 5.002 | 5.002 | 5.002 |
| WHO X | WGA |  |  | 37.001 | 37.001 | 37.001 | 37.001 |
|  | LA |  | 37.001 | 37.001 | 37.001 | 37.001 | 37.001 |

Supplemental table 6**. Detection of penA using de novo assemblies**. penA alleles were determined from each strain (Strain), detected from two different assembly approaches (Method), over a range of 5 different sub sampled depths (Depth N). Whole genome assembly (WGA) was performed with RA and local assemblies (LA) were performed with wtdbg2. Where identified the penA was correct.

| Sample name | Allele | AMR Markers | Beta-Lactamase |
| --- | --- | --- | --- |
| 202 | penA2.001 | penA Type II NonMosaic | Negative |
| 206 | penA43.002 | penA Type 43 NonMosaic; A502V | Positive |
| 250 | penA2.001 | penA Type II NonMosaic | Negative |
| 271 | penA43.002 | penA Type 43 NonMosaic; A502V | Positive |
| 294 | penA43.002 | penA Type 43 NonMosaic; A502V | Positive |
| 301 | penA5.002 | penA Type V NonMosaic | Negative |
| 304 | penA14.001 | penA Type XIV NonMosaic | Negative |
| 314 | penA2.001 | penA Type II NonMosaic | Negative |
| 315 | penA5.002 | penA Type V NonMosaic | Negative |

Supplemental table 7**, penA alleles identified by Nanopore from clinical metagenomic samples and validated with Illumina sequenced cultures**.
