# Supplementary material for: High precision Neisseria gonorrhoeae variant and antimicrobial resistance calling from metagenomic Nanopore sequencing
Source: Genome Res. 2020 Sep;30(9):1354–63. doi: 10.1101/gr.262865.120 (PMC7545138; doi:10.1101/gr.262865.120)
Supplement: Supplemental Material [file supp_gr.262865.120_Supplemental_Code.tar.zip › Supplemental_Code.tar.gz!/ngonpipe-master/threshold_analysis/images/SNP_locations.pdf]

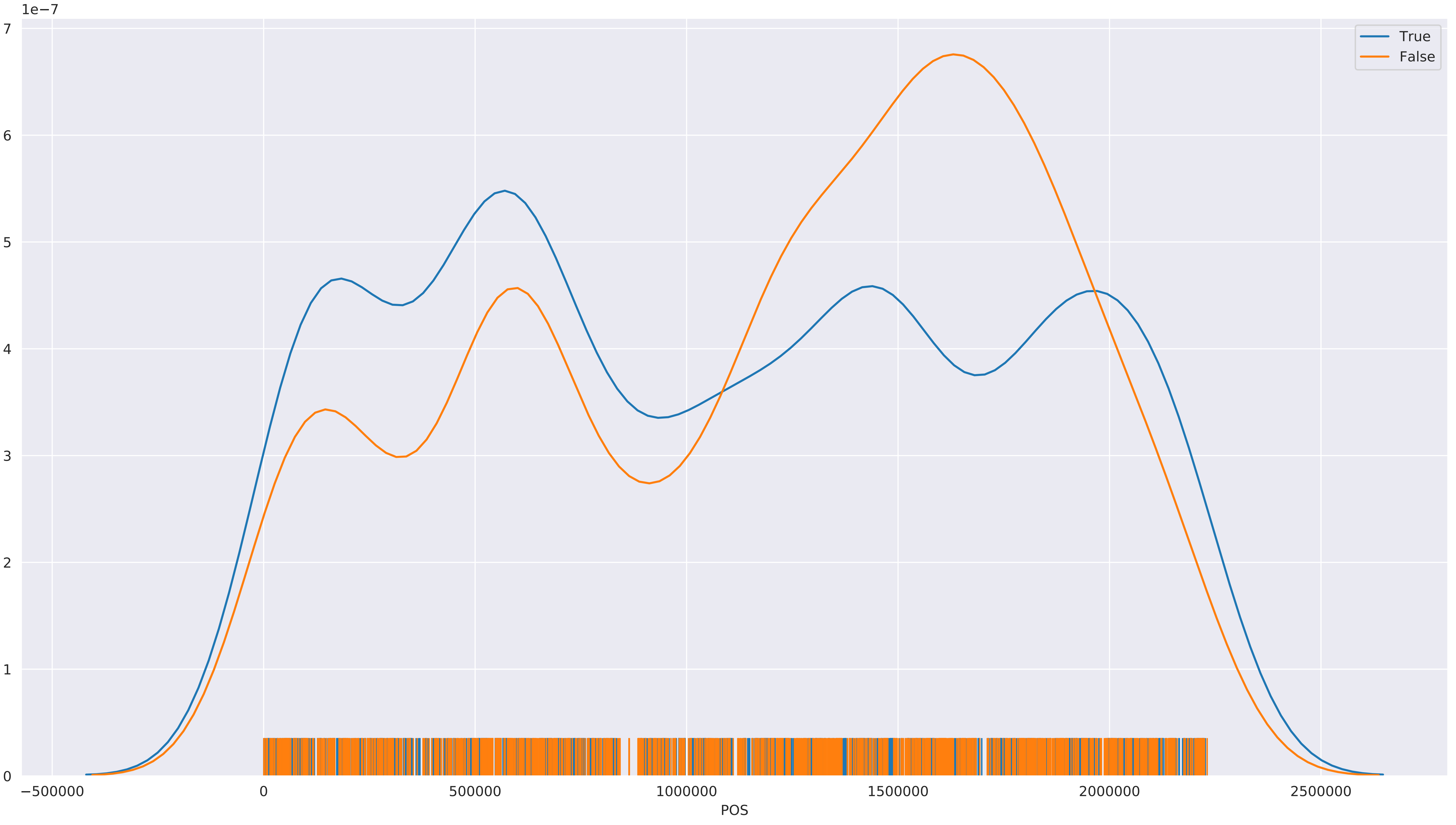
1e−7
7
6
5
4
3
2
1
0
−500000
0
500000
1000000
1500000
2000000
2500000
POS
True
False
