# Supplementary material for: High precision Neisseria gonorrhoeae variant and antimicrobial resistance calling from metagenomic Nanopore sequencing
Source: Genome Res. 2020 Sep;30(9):1354–63. doi: 10.1101/gr.262865.120 (PMC7545138; doi:10.1101/gr.262865.120)
Supplement: Supplemental Material [file supp_gr.262865.120_Supplemental_Code.tar.zip › Supplemental_Code.tar.gz!/ngonpipe-master/threshold_analysis/images/SNP_validated_qualts.pdf]

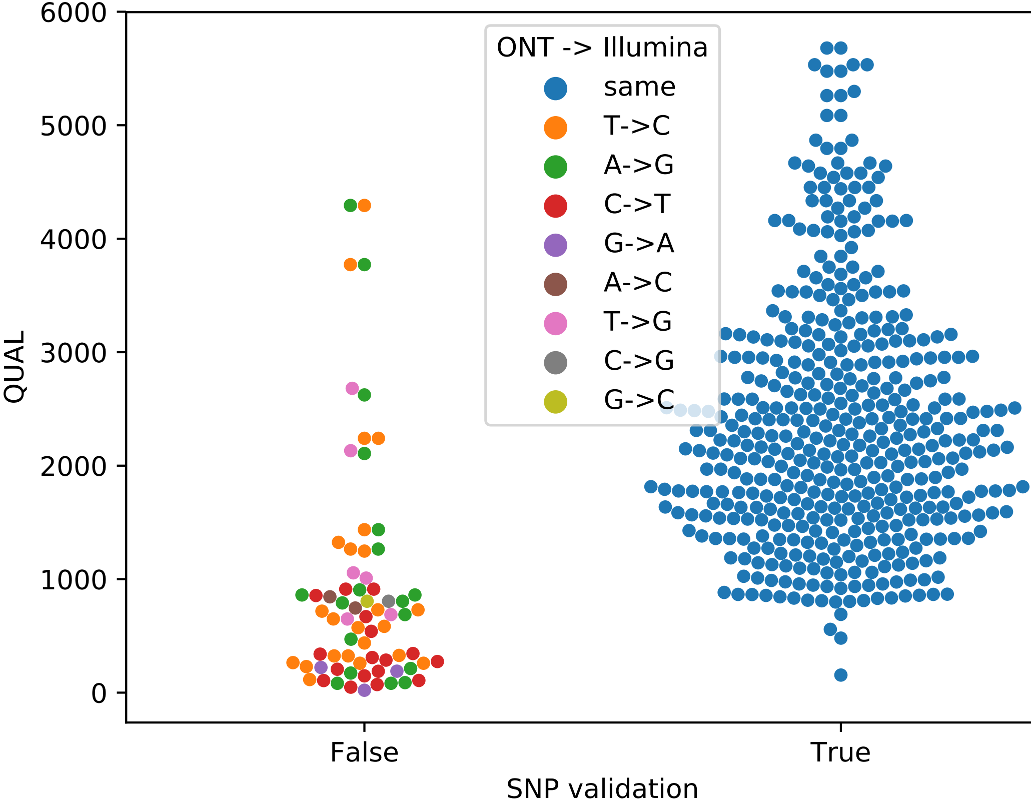
ONT -> Illumina
same
T->C
A->G
C->T
G->A
A->C
T->G
C->G
G->C
6000
5000
4000
3000
2000
1000
0
QUAL
False
True
SNP validation
