# Supplementary material for: High precision Neisseria gonorrhoeae variant and antimicrobial resistance calling from metagenomic Nanopore sequencing
Source: Genome Res. 2020 Sep;30(9):1354–63. doi: 10.1101/gr.262865.120 (PMC7545138; doi:10.1101/gr.262865.120)
Supplement: Supplemental Material [file supp_gr.262865.120_Supplemental_Code.tar.zip › Supplemental_Code.tar.gz!/ngonpipe-master/threshold_analysis/images/SNPs_over_supporting_reads.pdf]

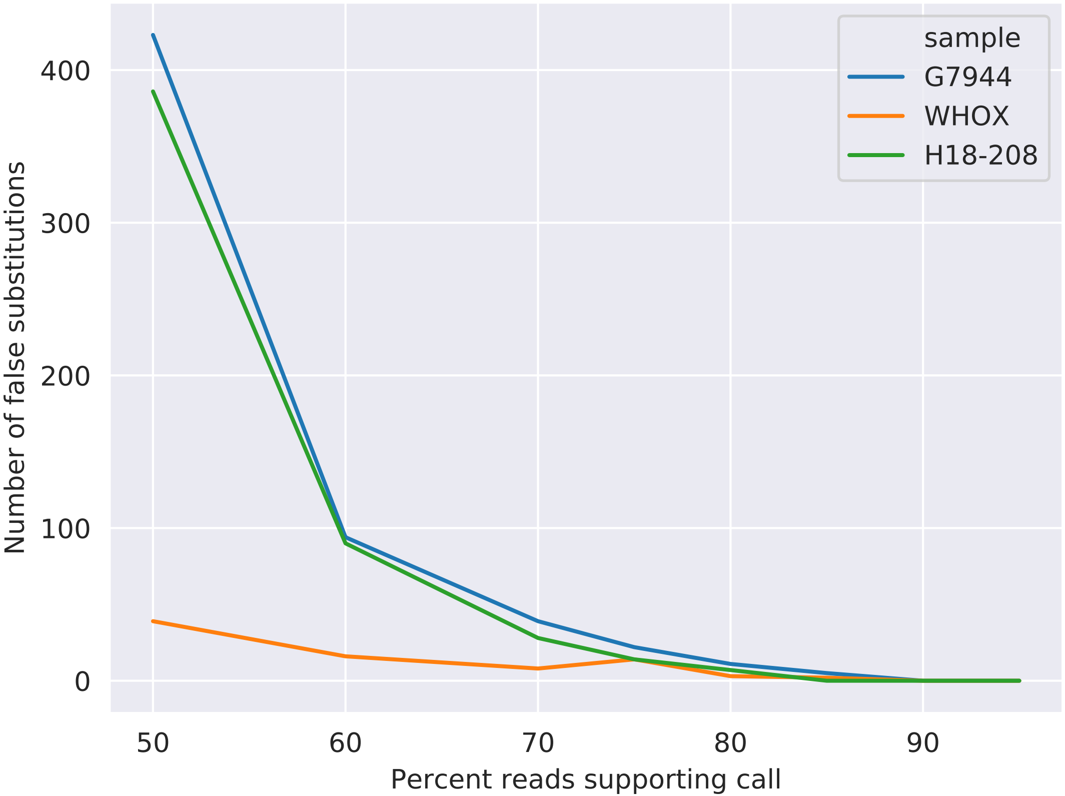

sample
G7944
WHOX
H18-208
400
300
200
100
0
50
60
70
80
90
Percent reads supporting call
Number of false substitutions
