# Supplementary material for: High precision Neisseria gonorrhoeae variant and antimicrobial resistance calling from metagenomic Nanopore sequencing
Source: Genome Res. 2020 Sep;30(9):1354–63. doi: 10.1101/gr.262865.120 (PMC7545138; doi:10.1101/gr.262865.120)
Supplement: Supplemental Material [file supp_gr.262865.120_Supplemental_Code.tar.zip › Supplemental_Code.tar.gz!/ngonpipe-master/threshold_analysis/images/indel_lengths_qual.pdf]

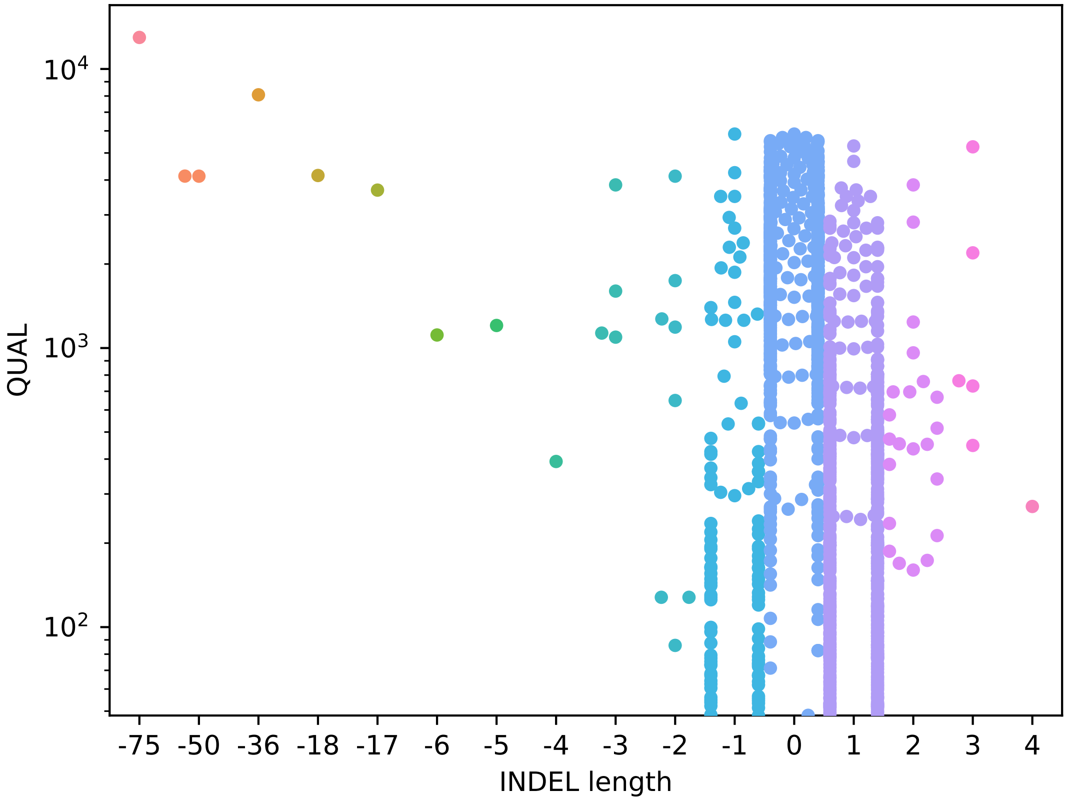

QUAL
10^4
10^3
10^2
-75
-50
-36
-18
-17
-6
-5
-4
-3
-2
-1
0
1
2
3
4
INDEL length
